# Individual components and cumulative burden of metabolic syndrome are associated with higher disease activity and adverse outcomes in Crohn’s disease

**DOI:** 10.3389/fmed.2025.1721566

**Published:** 2025-12-16

**Authors:** Shanshan Huang, Yuxia Pan, Zhudong Liu, Yanhua Liang, Shanyu Qin, Haixing Jiang

**Affiliations:** 1 Guangxi Medical University, Nanning, Guangxi, China; 2 Department of Gastroenterology, The First Affiliated Hospital of Guangxi Medical University, Nanning, Guangxi, China

**Keywords:** Crohn’s disease, metabolic syndrome, dyslipidemia, clinical outcomes, cumulative metabolic burden

## Abstract

**Background:**

The impact of metabolic syndrome (MetS) and its components on Crohn’s disease (CD) remains unclear. This study investigated how individual MetS factors and cumulative metabolic burden affect CD activity and outcomes.

**Methods:**

This retrospective study was conducted at a tertiary care hospital and encompassed a cohort of 376 hospitalized patients diagnosed with CD from 2015 to 2025. Linear, logistic, and Poisson regression models assessed correlations between MetS elements and clinical indicators, evaluating how their presence and number affected CD.

**Results:**

Thirty-six patients (9.6%) had MetS. Compared to non-MetS patients, those with MetS exhibited a significantly higher Simple Endoscopic Score for CD (10 vs. 7; *p* < 0.001), Crohn’s Disease Activity Index (294.3 vs. 256.6; *p* < 0.001), risks of complications (OR = 8.65, 95% CI: 2.01–37.26, *p* = 0.004) and surgery or invasive procedures (OR = 2.64, 95% CI: 1.27–5.45, *p* = 0.009). Low high-density lipoprotein cholesterol conferred a higher risk of adverse outcomes. The cumulative number of MetS elements exhibited an incremental effect, with increasing numbers correlating to progressively higher disease severity and a risk of poor outcomes.

**Conclusion:**

The concurrent presence of multiple MetS elements can synergistically worsen the clinical course of CD. Management of these components is crucial for the long-term prognosis of CD.

## Introduction

Crohn’s disease (CD) is a chronic condition that causes inflammation of the bowel. It often presents at a young age and is characterized by numerous complications, a prolonged disease course, and a high disability rate, severely impacting patients’ quality of life and long-term prognosis (1). Metabolic syndrome (MetS), characterized by obesity, hypertension, hyperglycemia, and dyslipidemia, is increasing in global prevalence (2). Individuals with MetS typically experience poor general health, potentially accelerating the progression of underlying conditions and increasing the risk of complications. MetS confers a risk of ischemic heart disease, stroke, and diabetes, and also promotes the progression of chronic kidney disease, hypertriglyceridemic acute pancreatitis, and various cancer (3–5). An intricate interplay between metabolic disturbances and intestinal inflammation is increasingly recognized (6). While most current studies focus on the impact of MetS on IBD incidence, systematic research on how MetS and its components affect CD severity and clinical outcomes is limited and inconclusive. This research is designed to explore the impact of all MetS elements and their cumulative number on disease activity and prognosis in CD.

## Materials and methods

### Study design

This single-center retrospective cohort study consecutively enrolled adult patients first diagnosed with CD at a comprehensive medical center between January 2015 and February 2025. Exclusion criteria included pregnancy, comorbid malignancy, severe hepatic or renal dysfunction, other major diseases affecting metabolic and inflammatory status, and incomplete clinical data. Ethical approval was obtained from the Institutional Review Board of The First Affiliated Hospital of Guangxi Medical University (2025-E0733).

### Definitions

Diagnosis of CD was established according to the clinical practice guidelines promulgated by the Organization of European Crohn’s and Colitis (ECCO), incorporating established clinical, endoscopic, radiological, and histological criteria (7). MetS components were defined using established Chinese diagnostic criteria (8), characterized by: hyperglycemia (fasting plasma glucose ≥6.1 mmol/L or a known diagnosis of diabetes); hypertension (blood pressure ≥130/85 mmHg or a known diagnosis of hypertension); hypertriglyceridemia (HTG) (TG levels ≥1.7 mmol/L); low high-density lipoprotein cholesterol (Low HDL-C) (HDL-C ≤1.0 mmol/L); and obesity, characterized specifically by a body mass index (BMI) ≥28 kg/m^2^ rather than waist circumference. Patients with three or more of these components were diagnosed with MetS.

### Data collection

Two trained researchers independently collected data, including demographic, endoscopic, and laboratory data from electronic medical records using standardized data extraction forms. All extracted data were reviewed by a clinical gastroenterology specialist. Collected data included demographic characteristics (age, sex, smoking history, alcohol history), MetS-related indicators (history of diabetes and hypertension, admission blood pressure, fasting glucose, TG, HDL-C, BMI), nutritional status parameters (serum albumin, prealbumin), inflammatory and immune markers [C-reactive protein (CRP), erythrocyte sedimentation rate (ESR), fecal calprotectin], disease characteristics and clinical scores [Montreal classification behavior and location, Crohn’s Disease Activity Index (CDAI), Simple Endoscopic Score for Crohn’s Disease (SES-CD)], clinical outcomes and complications (intestinal complications such as stenosis, obstruction, perforation, bleeding, or abscess; perianal complications including abscess or fistula; other fistulas; extraintestinal manifestations such as pyoderma gangrenosum; malignancy), treatments and interventions (intestinal surgery, perianal surgery, biologic use), and other outcome measures (malnutrition, antibiotic use, hospitalization duration and costs, and mortality). Except for long-term outcomes such as complications, surgical history, and nutritional status that covered the entire disease course, other metabolic and inflammatory indicators were obtained from the data during the patient’s first diagnosis at our hospital to reflect the objective state before standardized diagnosis and treatment.

### Statistical analysis

Normally distributed continuous variables are reported as mean ± standard deviation, with group comparisons performed using Student’s *t*-test. Non-normally distributed data are summarized as median (interquartile range) and compared using non-parametric tests, including the Mann–Whitney *U* test and the Kruskal–Wallis test. Categorical variables are presented as frequency (percentage) and compared using *χ*^2^ or Fisher’s exact tests. Linear and logistic regression models were used to analyze associations between MetS elements and clinical indicators or outcomes, adjusted for confounders such as age and sex. Linear trends among ordered groups were evaluated by means of the Mantel–Haenszel method. Correlations between ordinal categorical variables were further quantified using Spearman’s rank correlation and Poisson regression. Statistical analyses were carried out with SPSS Statistics (version 26.0). Graphical representations were created using GraphPad Prism (version 9.5). *p* < 0.05 indicates statistical significance.

## Results

### Overall patient characteristics

A cohort of 376 patients with CD was enrolled in this study. The general characteristics of the study cohort are presented in Table 1. Among the patients, 279 were male (74.2%) and 97 were female (25.8%), with a median age of 32 (23, 45) years. The median BMI was 18.42 (16.66, 20.93) kg/m^2^. Thirty-six patients (9.6%) had MetS, and 257 (68.4%) had low HDL-C. 236 patients (62.8%) had ileocolonic disease, 177 (47.1%) had non-stricturing non-penetrating behavior. The median CDAI score was 257 (220, 297.5), indicating moderate disease activity. 255 patients (68.1%) had complications. 122 patients (32.4%) underwent surgery or invasive procedures due to primary disease, and 51 (13.6%) underwent intestinal resection. 309 patients (82.2%) were treated with biologics. Due to limited data on pyoderma gangrenosum, malignancy, and mortality, these parameters were not further analyzed.

**Table 1:** Patient characteristics, median (IQR), *n* (%).

Characteristic	All (*n* = 376)
Age, years	32 (23, 45)
Sex	
Male	279 (74.2%)
Female	97 (25.8%)
Smoking history	96 (25.5%)
History of alcohol	90 (23.9%)
BMI, kg/m^2^	18.42 (16.66, 20.93)
Mets	36 (9.6%)
Obesity	3 (0.79%)
Hypertension	77 (20.5%)
Hyperglycemia	20 (5.3%)
HTG	76 (20.2%)
Low HDL-C	257 (68.4%)
SES-CD	7 (5, 7)
CDAI	257 (220, 297.5)
Fecal calprotectin	587 (345.3, 809.6)
Disease behavior	
Non-stricturing, non-penetrating	177 (47.1%)
Stricturing	147 (39.1%)
Penetrating	22 (5.9%)
Stricturing with penetrating	30 (8%)
Disease location	
Ileum	73 (19.4%)
Colon	61 (16.2%)
Ileocolonic	236 (62.8%)
Upper gastrointestinal tract	6 (1.6%)
Complications	255 (68.1%)
Intestinal stenosis	170 (45.2%)
Intestinal obstruction	76 (20.2%)
Perforation	15 (3.9%)
Gastrointestinal bleeding	41 (10.9%)
Intra-abdominal abscess	16 (4.3%)
Perianal abscess	69 (18.4%)
Anal fissure	3 (0.8%)
Anal fistula	70 (18.6%)
Other fistulas	29 (7.7%)
Pyoderma gangrenosum	1 (0.3%)
Crohn’s-related intestinal carcinoma	1 (0.3%)
Surgical or invasive procedures	122 (32.4%)
Intestinal resection	51 (13.6%)
Fistula-related surgery	35 (9.3%)
Perianal abscess surgery	37 (9.8%)
Intra-abdominal abscess surgery	5 (1.3%)
Perforation repair	5 (1.3%)
Intestinal adhesiolysis	3 (0.8%)
Biologic therapy	309 (82.2%)
Length of hospital stays, days	9 (7, 9)
Total costs, thousand CNY	13.6 (10.5, 18.2)
Mortality	2 (0.53%)

IQR, interquartile range; BMI, body mass index; MetS, metabolic syndrome; HTG, hypertriglyceridemia; Low HDL-C, low high-density lipoprotein cholesterol; SES-CD, Simplified Endoscopic Score for Crohn’s disease; CDAI, Crohn’s Disease Activity Index; CNY, Chinese Yuan.

### Metabolic syndrome components and their associations with laboratory parameters, clinical scores, and hospitalization profile

**Figure 1:**
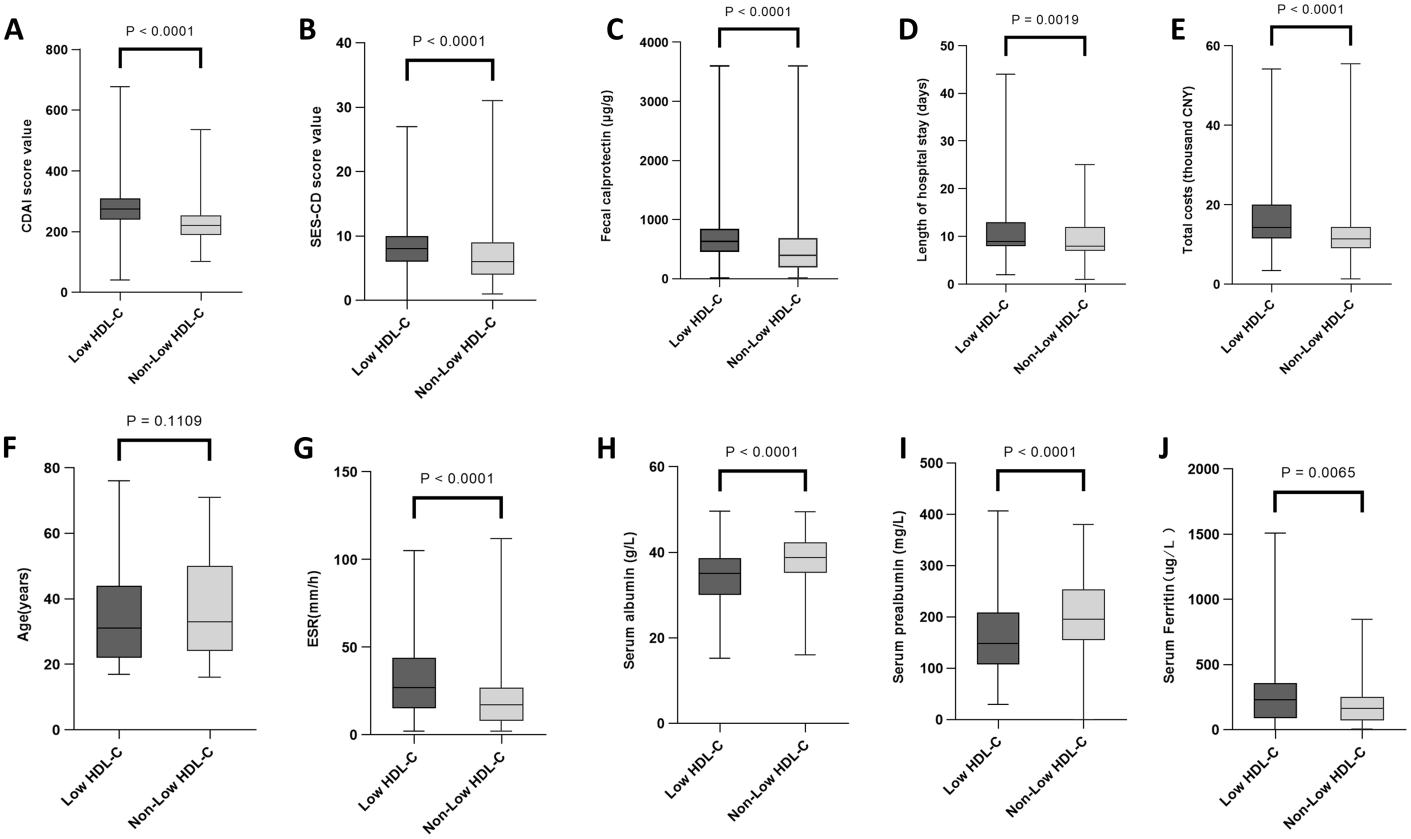
Associations of low high-density lipoprotein cholesterol with laboratory parameters, clinical scores, and hospitalization characteristics in Crohn’s disease. Comparison between patients with and without low HDL-C. **(A)** CDAI. **(B)** SES-CD. **(C)** Fecal calprotectin. **(D)** Length of hospital stay. **(E)** Total costs. **(F)** Age. **(G)** ESR. **(H)** Serum albumin. **(I)** Serum prealbumin. **(J)** Serum ferritin. Low HDL-C, low high-density lipoprotein cholesterol; ESR, erythrocyte sedimentation rate; SES-CD, Simple Endoscopic Score for Crohn’s disease; CDAI, Crohn’s Disease Activity Index; CNY, Chinese Yuan. Data are presented as median and interquartile range. The Mann–Whitney *U* test was used for comparisons of continuous variables.

**Figure 2:**
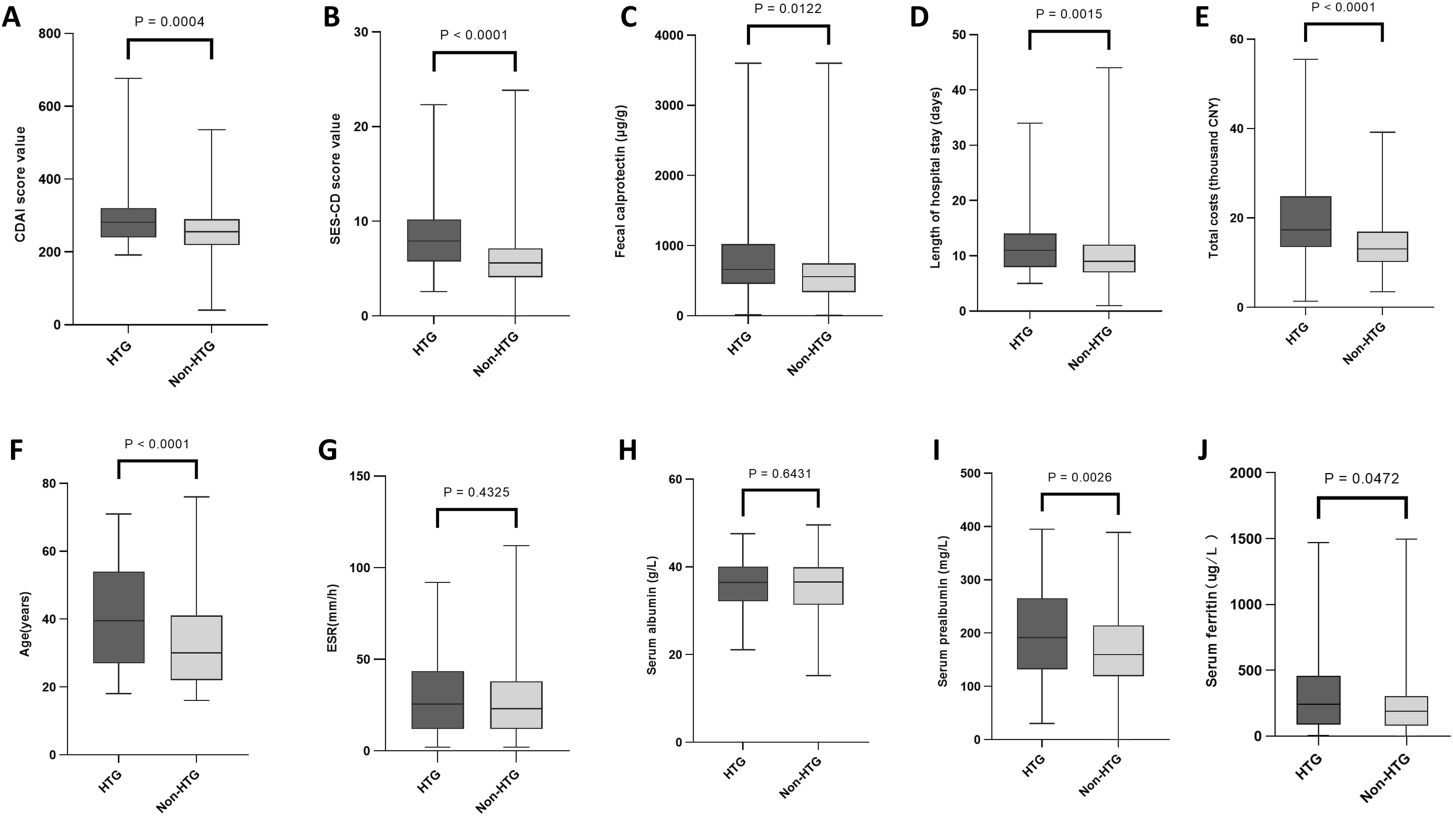
Associations of hypertriglyceridemia with laboratory parameters, clinical scores, and hospitalization characteristics in Crohn’s disease. Comparison between patients with and without hypertriglyceridemia. **(A)** CDAI. **(B)** SES-CD. **(C)** Fecal calprotectin. **(D)** Length of hospital stay. **(E)** Total costs. **(F)** Age. **(G)** ESR. **(H)** Serum albumin. **(I)** Serum prealbumin. **(J)** Serum ferritin. HTG, hypertriglyceridemia; ESR, erythrocyte sedimentation rate; SES-CD, Simple Endoscopic Score for Crohn’s disease; CDAI, Crohn’s Disease Activity Index; CNY, Chinese Yuan. Data are presented as median and interquartile range. The Mann–Whitney *U* test was used for comparisons of continuous variables.

**Figure 3:**
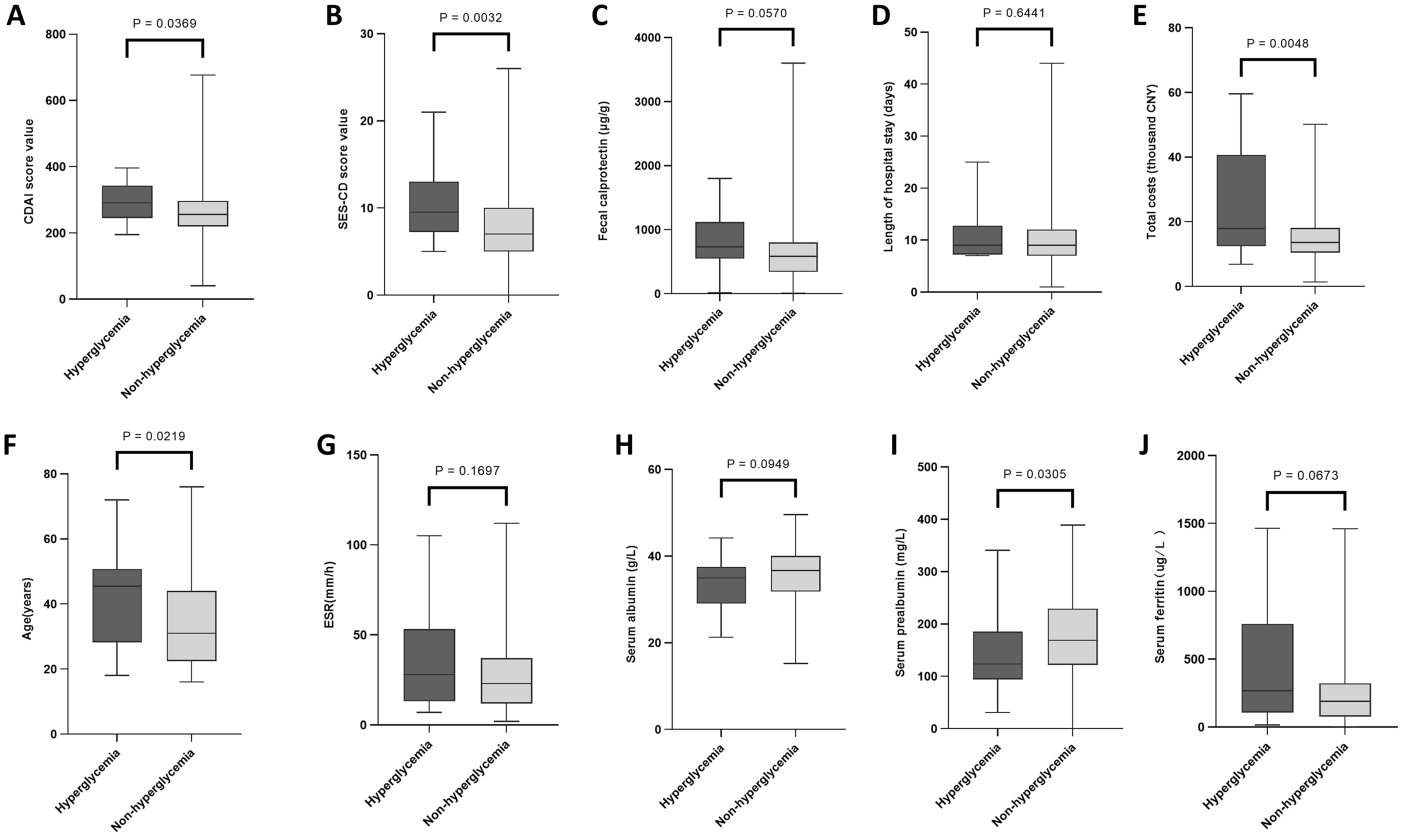
Associations of hyperglycemia with laboratory parameters, clinical scores, and hospitalization characteristics in Crohn’s disease. Comparison between patients with and without hyperglycemia. **(A)** CDAI. **(B)** SES-CD. **(C)** Fecal calprotectin. **(D)** Length of hospital stay. **(E)** Total costs. **(F)** Age. **(G)** ESR. **(H)** Serum albumin. **(I)** Serum prealbumin. **(J)** Serum ferritin. ESR, erythrocyte sedimentation rate; SES-CD, Simple Endoscopic Score for Crohn’s disease; CDAI, Crohn’s Disease Activity Index; CNY, Chinese Yuan. Data are presented as median and interquartile range. The Mann–Whitney *U* test was used for comparisons of continuous variables.

**Figure 4:**
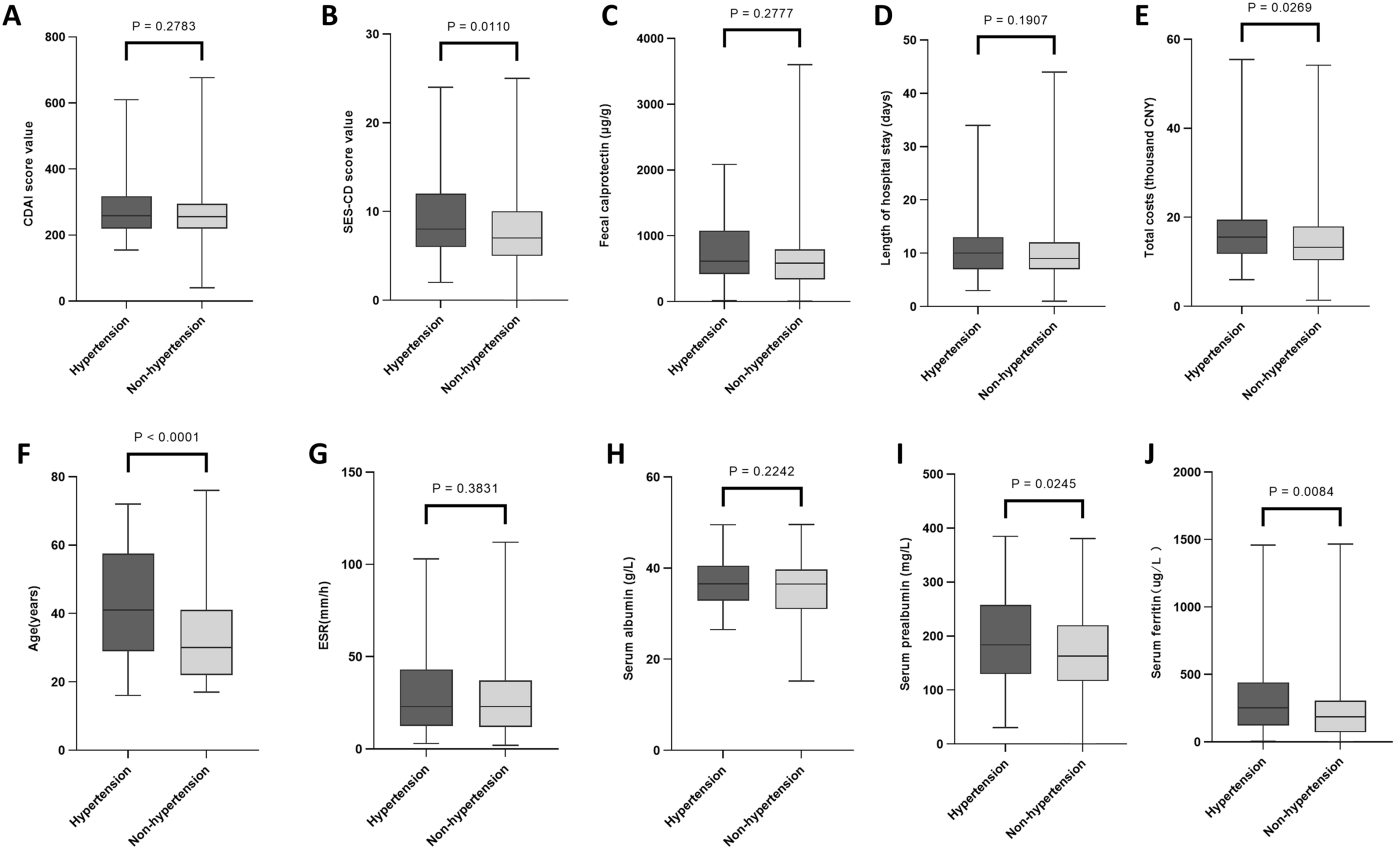
Associations of hypertension with laboratory parameters, clinical scores, and hospitalization characteristics in Crohn’s disease. Comparison between patients with and without hypertension. **(A)** CDAI. **(B)** SES-CD. **(C)** Fecal calprotectin. **(D)** Length of hospital stay. **(E)** Total costs. **(F)** Age. **(G)** ESR. **(H)** Serum albumin. **(I)** Serum prealbumin. **(J)** Serum ferritin. ESR, erythrocyte sedimentation rate; SES-CD, Simple Endoscopic Score for Crohn’s disease; CDAI, Crohn’s Disease Activity Index; CNY, Chinese Yuan. Data are presented as median and interquartile range. The Mann–Whitney *U* test was used for comparisons of continuous variables.

As presented in Figures 1–4, patients were stratified into two cohorts according to whether they exhibited low HDL-C, HTG, hyperglycemia, and hypertension. Low HDL-C was correlated with higher levels of inflammatory markers (ESR, fecal calprotectin), more severe disease activity (SES-CD, CDAI), higher medical costs, and lower nutritional indicators (albumin, prealbumin). Hypertriglyceridemia was associated with older age, higher disease activity, and higher hospitalization costs. Hyperglycemia was correlated with higher disease activity, hospitalization costs, and older age. Hypertension was correlated with older age and higher hospitalization costs. Detailed data are presented in Supplementary Table 1.

Multiple linear regression analysis revealed that higher BMI showed protective associations (Table 2). After adjustment for common confounding factors, each 1 kg/m^2^ increment in BMI was significantly correlated with the following changes: a 0.64 g/L increase in serum albumin (95% CI: 0.17 to 1.12; *p* = 0.008), an 8.34 mg/L elevation in serum prealbumin (95% CI: 4.02 to 12.66; *p* < 0.001), a 28.93 μg/g reduction in fecal calprotectin (95% CI: −47.92 to −9.93; *p* = 0.003), a 5.52-point decrease in CDAI score (95% CI: −7.84 to −3.20; *p* < 0.001), and a 0.18-day shortening of hospital stay (95% CI: −0.34 to −0.01; *p* = 0.034).

**Table 2:** Relationship between body mass index and laboratory indicators, clinical scores, and hospitalization characteristics in Crohn’s disease.

Outcomes	*B*	95% CI	*p-*value
ESR, mm/h	−0.50	−0.184, 0.192	0.157
Serum albumin, g/L	0.64	0.17, 1.12	0.008^a^
Serum prealbumin, mg/L	8.34	4.02, 12.66	<0.001^a^
Serum ferritin, ug/L	−1.6	−15.2, 12	0.817
Fecal calprotectin, μg/g	−28.93	−47.92, −9.93	0.003^a^
SES-CD	−0.2	−0.345, −0.056	0.007^a^
CDAI	−5.52	−7.84, −3.20	<0.001^a^
Age, years	0.82	0.40, 1.24	<0.001^a^
Length of hospital stay, days	−0.18	−0.34, −0.01	0.034^a^
Total costs, thousand CNY	−0.84	−3.82, 2.14	0.578

a *p* < 0.05.

*B*, regression coefficient; CI, confidence interval; IQR, interquartile range; ESR, erythrocyte sedimentation rate; SES-CD, Simplified Endoscopic Score for Crohn’s disease; CDAI, Crohn’s Disease Activity Index; CNY, Chinese Yuan.

Models were adjusted for age, sex, smoking history, and alcohol use. Linear regression models were used for analysis.

### Impact of individual components of metabolic syndrome on clinical outcomes in patients

Binary logistic regression analysis was employed to explore the association between low HDL-C, HTG, hypertension, hyperglycemia, BMI, and adverse clinical outcomes in CD patients. Table 3 shows that low HDL-C patients had increased risks of complications (OR = 12.09, 95% CI: 7.12–20.6, *p* < 0.001), elevated CRP (OR = 2.98, 95% CI: 1.73–5.12, *p* < 0.001), antibiotic use (OR = 2.07, 95% CI: 1.19–3.59, *p* = 0.01), malnutrition (OR = 2.15, 95% CI: 1.12–4.12, *p* = 0.021), and surgery or procedures (OR = 3.28, 95% CI: 1.87–5.73, *p* < 0.001). Patients with hypertriglyceridemia had increased risks of complications (OR = 4.12, 95% CI: 1.99–8.52, *p* < 0.001) and surgery or procedures (OR = 1.95, 95% CI: 1.14–3.34, *p* = 0.015). Patients with hyperglycemia had increased risks of malnutrition (OR = 4.93, 95% CI: 1.78–13.66, *p* = 0.002) and surgery or procedures (OR = 2.56, 95% CI: 1.00–6.50, *p* = 0.049). Higher BMI was associated with a reduced risk of malnutrition (OR = 0.66, 95% CI: 0.58–0.76, *p* < 0.001), elevated CRP (OR = 0.88, 95% CI: 0.81–0.96, *p* = 0.002), intestinal stricture (OR = 0.93, 95% CI: 0.86–0.99, *p* = 0.024), intestinal obstruction (OR = 0.88, 95% CI: 0.80–0.96, *p* = 0.007), and antibiotic use (OR = 0.90, 95% CI: 0.83–0.97, *p* = 0.009). No statistically significant differences in these measures were found when comparing the hypertensive and non-hypertensive groups. Patients with MetS had increased risks of complications (OR = 8.65, 95% CI: 2.01–37.26, *p* = 0.004) and surgery or invasive procedures (OR = 2.64, 95% CI: 1.27–5.45, *p* = 0.009).

**Table 3:** Impact of individual metabolic syndrome elements on adverse clinical outcomes in Crohn’s disease.

Outcomes	Low HDL-C		HTG		Hypertension		Hyperglycemia		BMI		Mets	
	OR (95%CI)	*p-*value	OR (95%CI)	*p-*value	OR (95%CI)	*p-*value	OR (95%CI)	*p-*value	OR (95%CI)	*p-*value	OR (95%CI)	*p-*value
Complications	12.09 (7.12, 20.60)	<0.001^a^	4.12 (1.99, 8.52)	<0.001^a^	0.89 (0.49, 1.59)	0.684	3.08 (0.86, 11.00)	0.083	0.94 (0.88, 1.00)	0.082	8.65 (2.01, 37.26)	0.004^a^
Elevated CRP	2.98 (1.73, 5.12)	<0.001^a^	1.10 (0.57, 2.14)	0.779	1.59 (0.76, 3.33)	0.222	4.92 (0.64, 37.93)	0.127	0.88 (0.81, 0.96)	0.002^a^	1.55 (0.56, 4.25)	0.396
Intestinal stenosis	5.26 (3.10, 8.94)	<0.001^a^	1.62 (1.32, 2.74)	0.04^a^	0.85 (0.49, 1.47)	0.567	0.81 (0.31, 2.08)	0.654	0.93 (0.86, 0.99)	0.024^a^	1.51 (0.73, 3.11)	0.266
Intestinal obstruction	3.60 (0.73, 3.11)	0.001^a^	2.12 (1.17, 3.85)	0.013^a^	0.77 (0.39, 1.51)	0.444	0.36 (0.08, 1.64)	0.185	0.88 (0.80, 0.96)	0.007^a^	0.92 (0.39, 2.16)	0.847
Intestinal perforation	8.78 (1.10, 70.00)	0.04^a^	3.08 (1.05, 9.05)	0.041^a^	1.38 (0.33, 5.67)	0.659	1.14 (1.14, 9.53)	0.902	1.05 (0.88, 1.24)	0.578	4.50 (1.33, 15.2)	0.016^a^
Gastrointestinal bleeding	3.14 (1.26, 7.82)	0.014^a^	1.57 (0.75, 3.32)	0.235	0.71 (0.29, 1.71)	0.443	1.25 (0.33, 4.54)	0.740	1.05 (0.95, 1.16)	0.379	1.13 (0.40, 3.20)	0.822
Perianal abscess	2.18 (1.01, 4.36)	0.028^a^	1.13 (0.40, 3.20)	0.822	1.48 (0.71, 3.08)	0.299	2.19 (0.61, 7.81)	0.227	1.09 (0.99, 1.18)	0.054	2.61 (1.04, 6.58)	0.042^a^
Anal fistula	2.97 (1.43, 6.15)	0.003^a^	1.75 (0.89, 3.45)	0.104	1.33 (0.65, 2.74)	0.435	1.19 (0.31, 4.55)	0.797	1.04 (0.961.13)	0.349	2.35 (0.96, 5.75)	0.061
Other fistulas	3.24 (1.08, 9.77)	0.036^a^	0.63 (0.23, 1.76)	0.379	0.81 (0.30, 2.15)	0.668	1.86 (0.49, 6.99)	0.358	0.87 (0.76, 1.00)	0.055	0.75 (0.21, 2.78)	0.675
Antibiotic use	2.07 (1.19, 3.59)	0.010^a^	0.86 (0.47, 1.55)	0.609	1.05 (0.58, 1.91)	0.875	1.83 (0.71, 4.70)	0.208	0.90 (0.83, 0.97)	0.009^a^	1.55 (0.74, 3.26)	0.250
Malnutrition	2.15 (1.12, 4.12)	0.021^a^	0.55 (0.25, 1.43)	0.148	0.58 (0.25, 1.32)	0.197	4.93 (1.78, 13.66)	0.002^a^	0.66 (0.58, 0.76)	<0.001^a^	0.95 (0.34, 2.63)	0.919
Surgical or invasive procedures	3.28 (1.87, 5.73)	<0.001^a^	1.95 (1.14, 3.34)	0.015^a^	1.28 (0.73, 2.26)	0.392	2.56 (1.00, 6.50)	0.049^a^	1.02 (0.95, 1.09)	0.602	2.64 (1.27, 5.45)	0.009^a^
Intestinal resection	3.78 (1.60, 8.93)	0.002^a^	2.11 (1.09, 4.09)	0.027^a^	1.34 (0.65, 2.76)	0.434	3.07 (1.13, 8.35)	0.028^a^	0.94 (0.85, 1.04)	0.223	1.81 (1.76, 4.27)	0.022^a^
Fistula surgery	3.32 (1.10, 9.55)	0.034^a^	1.45 (0.60, 3.48)	0.404	1.27 (0.76, 3.22)	0.619	1.51 (0.31, 7.37)	0.611	1.11 (0.99, 1.23)	0.061	1.94 (0.66, 5.73)	0.231

a *p* < 0.05.

OR, odds ratio; CI, confidence interval; Low HDL-C, low high-density lipoprotein cholesterol; HTG, hypertriglyceridemia; BMI, body mass index; MetS, metabolic syndrome; CRP, C-reactive protein.

Models were adjusted for age, sex, smoking history, and alcohol use. Binary logistic regression models were used for analysis.

### Association of the number of metabolic syndrome components with clinical scores and hospitalization characteristics in patients

To investigate how the cumulative number of metabolic syndrome elements affects the disease trajectory of CD, patients were divided into five cohorts corresponding to the presence of 0 to 4 MetS features. As shown in Table 4, the Kruskal–Wallis test indicated highly statistically significant differences across groups for all observed indicators. More importantly, linear regression analysis revealed a burden-response relationship: an increasing number of MetS elements was significantly associated with worse clinical outcomes, including elevated disease activity (SES-CD, CDAI), higher inflammatory marker levels (ESR, serum ferritin, fecal calprotectin), and extended hospital stays (*p* < 0.001). The burden of complications escalated commensurately with the increasing number of MetS components. Spearman correlation analysis confirmed a significant positive correlation (*ρ* = 0.362, *p* < 0.001). A Poisson regression model adjusted for potential confounders quantified this relationship, indicating that each additional MetS component was correlated with a 33% average increase in the number of complications (IRR = 1.33, 95% CI: 1.21–1.47, *p* < 0.001).

**Table 4:** Relationship between the number of metabolic syndrome elements and laboratory indicators, clinical scores, and hospitalization characteristics in Crohn’s disease, Median (IQR).

	Cumulative number of metabolic syndrome elements							
Characteristic	0 (*n* = 79)	1 (*n* = 200)	2 (*n* = 62)	3 (*n* = 32)	4 (*n* = 3)	*p-*value	B (95% CI)	*p-*value
ESR, mm/h	16 (7, 24)	25 (14, 40)	24 (14, 40)	29 (10, 66)	42 (27, 58)	<0.001^a^	5.29 (2.72, 7.87)	<0.001^a^
Serum albumin, g/L	38.80 (35.10, 42.00)	35.70 (30.50, 38.90)	35.80 (32.10, 39.90)	36.3 (31.40, 39.60)	29 (28, 33)	0.001^a^	0.09 (−1.63, 1.81)	0.92
Serum prealbumin, mg/L	200 (156, 256)	148 (111, 195)	184 (121, 138)	199 (126, 270)	91 (62, 107)	<0.001^a^	−10.11 (−25.83, 5.62)	0.21
Serum ferritin, ug/L	152.20 (80.60, 217.60)	215.10 (79.70, 328.80)	282.40 (61.60, 328.50)	250.30 (107.0, 451.5)	877.30 (828.20, 1543.50)	<0.001^a^	95.13 (44.15, 146.11)	<0.001^a^
Fecal calprotectin, μg/g	347.30 (178.20, 587.00)	611.30 (395.50, 790.30)	667.90 (486.60, 982.20)	629.60 (444.80, 1165.90)	1125.80 (1011.90, 1259.30)	<0.001^a^	110.65 (38.17, 183.13)	<0.001^a^
SES-CD	5 (4, 7)	7 (5, 9)	9 (6, 13)	10 (8, 14)	12 (11, 16.5)	<0.001^a^	1.83 (1.31, 2.36)	<0.001^a^
CDAI	211.67 (181.50, 243.20)	265.50 (228.00, 297.90)	270.60 (231.60, 317)	291.10 (246.40, 320.00)	335.20 (314.60, 347.90)	<0.001^a^	25.01 (16.37, 33.83)	<0.001^a^
Length of hospital stay, days	8 (6, 10)	9 (7, 12)	9 (8, 12)	12 (9, 15)	12 (11, 15)	<0.001^a^	1.19 (0.57, 1.80)	<0.001^a^
Total costs, thousand CNY	11.30 (8.80, 13.70)	13.60 (10.60, 18.40)	15.80 (11.70, 19.80)	17.30 (14.00, 27.40)	27.70 (22.50, 35.30)	<0.001^a^	221.13 (−493.81, 936.067)	0.543
Number of complications	0 (0, 1)	1 (0, 2)	2 (1, 2)	2 (1, 2)	3 (2, 3)	<0.001^a^	—	—
Age, years	31 (24, 40)	30 (21, 40)	37 (28, 49)	49 (28, 58)	58 (41, 59)	<0.001^a^	3.48 (1.89, 5.06)	<0.001^a^

a *p* < 0.05.

*B*, regression coefficient; CI, confidence interval; IQR, interquartile range; ESR, erythrocyte sedimentation rate; SES-CD, Simplified Endoscopic Score for Crohn’s disease; CDAI, Crohn’s Disease Activity Index; CNY, Chinese Yuan.

Kruskal–Wallis test and linear regression analysis were used for analysis. —, not applicable (linear regression is inappropriate for ordinal categorical data).

### Metabolic syndrome components demonstrate a cumulative impact on specific adverse outcomes in Crohn’s disease

**Figure 5:**
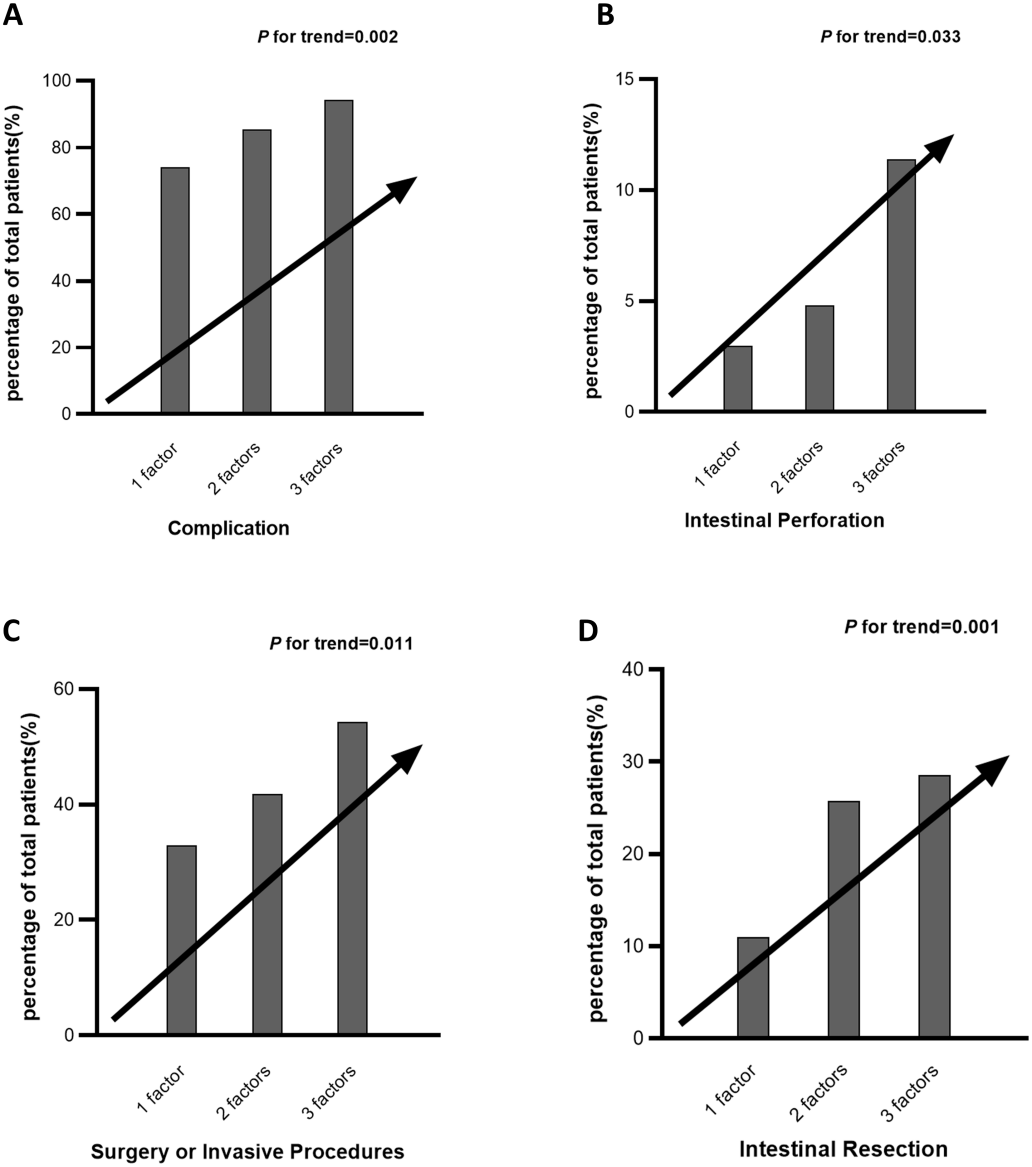
Cumulative impact of metabolic syndrome components on specific adverse outcomes in Crohn’s disease. Panels **A–D** illustrate the percentage of Crohn’s disease patients experiencing different adverse outcomes—including complications **(A)**, intestinal perforation **(B)**, surgery or invasive procedures **(C)**, and intestinal resection **(D)**—stratified by the number of metabolic syndrome components (1, 2, or 3). Patients with four metabolic components (*n* = 3) were pooled with those having three for analysis.

To assess the presence of a linear trend with increasing counts of MetS elements and the incidence of specific adverse outcomes, a Cochran–Mantel–Haenszel test for trend was conducted (Patients with four metabolic components (*n* = 3) were pooled with those having three for analysis). As shown in Figure 5, a linear elevation in risk for complications (*p* = 0.002), intestinal perforation (*p* = 0.033), surgical intervention (*p* = 0.011), and intestinal resection (*p* = 0.004) was observed with a higher number of MetS elements.

## Discussion

Our analysis revealed that specific elements of MetS, notably low HDL-C and HTG, were independently associated with more severe disease activity and worse clinical outcomes in Crohn’s disease. A significant dose–response relationship was observed for this association. The prevalence of MetS in this CD cohort (9.6%) was consistent with previous reports (9, 10), and was characterized by a distinct phenotype of “low obesity rate but high dyslipidemia,” with a notably high prevalence of low HDL-C (68.4%). This may be related to the context of malnutrition associated with CD, suggesting that even in underweight patients, dyslipidemia may serve as an important marker of disease severity.

Low HDL-C serves as a robust predictor of poor clinical outcomes in patients with CD (OR for complications = 12.09, *p* < 0.001). Mendelian randomization evidence suggests that low HDL-C is not merely a consequence of CD but may play a causative role in its pathogenesis (11). Integrating our present findings with previously established mechanisms, we propose a self-perpetuating pathogenic loop centered around HDL-C dysfunction: genetically or environmentally induced reduction or functional impairment of HDL-C compromises intestinal barrier integrity and anti-inflammatory capacity, generating a susceptible mucosal microenvironment. Upon inflammatory activation, cytokines and epigenetic regulators such as microRNA-33 collectively suppress the expression of ATP-binding cassette transporter A1 (ABCA1), further diminishing local HDL synthesis. Concurrently, microRNA-33 impairs mitochondrial metabolism in macrophages, attenuating their capacity to resolve inflammation. The resulting chronic inflammatory milieu exacerbates HDL consumption and functional degradation, ultimately leading to impaired mucosal healing and sustained disease activity. This model underscores the dual role of HDL-C in CD pathophysiology and highlights the therapeutic potential of strategies aimed at restoring HDL levels or functionality to disrupt this vicious cycle (12, 13). A recent retrospective study identified hypertriglyceridemia as a significant risk factor for increased hospitalization among patients with CD (13). Our study further delineates this association by specifying complications such as intestinal strictures and perforations, thereby extending the evidence regarding the impact of dyslipidemia on CD-related outcomes. HTG frequently coexists with low HDL-C, and both act synergistically to promote inflammatory progression. Normally, HDL-C can buffer lipotoxicity by binding free fatty acids; low HDL-C loses this capacity, leading to accumulation of free fatty acids in the intestinal mucosa, inducing endoplasmic reticulum stress and pyroptosis, and exacerbating barrier disruption (13). Our findings of a significant association between dyslipidemia and CD find strong corroboration in the established link between metabolic dysfunction-associated steatotic liver disease (MASLD) and IBD. MASLD is the most recent term replacing non-alcoholic fatty liver disease (NAFLD), previously known as metabolic dysfunction-associated fatty liver disease (MAFLD), reflecting a shift in definition towards emphasizing underlying metabolic dysfunction. The markedly higher prevalence of MASLD in IBD patients compared to the general population reveals a shared pathophysiological basis beyond coincidence (14, 15). Central to this association is a chronic, low-grade systemic inflammatory state, often termed “meta-inflammation,” and a critically important bridge—the gut microbiota. Both IBD and MASLD can be viewed as diseases driven by dysbiosis, characterized by the depletion of beneficial microbes and the enrichment of pro-inflammatory species. This dysbiosis compromises intestinal barrier integrity, leading to the translocation of microbial products such as lipopolysaccharide, which in turn can activate inflammatory cascades via pathways like Toll-like receptor signaling, driving metabolic dysfunction-associated steatohepatitis (MASH) in the liver and IBD inflammation in the gut mucosa (16). In our study, lipid abnormalities such as low HDL-C and HTG are not only diagnostic components of MetS and MASLD but may also collectively contribute to CD progression by exacerbating inflammation along this gut-liver axis. Consistent with prior research, hyperglycemia was clearly associated with malnutrition and increased surgical risk. Microvascular complications and immune dysfunction resulting from hyperglycemia may impede tissue repair and increase infection risk, thereby worsening the CD course (17).

In contrast to these clearly detrimental metabolic components, our study also revealed more complex associations. Higher BMI showed a “protective” association with better nutritional status and lower disease activity. This “obesity paradox” may be explained by sufficient nutritional reserves buffering inflammatory catabolism, or reverse causality whereby improved disease control leads to weight gain (18). It should be noted, however, that only 3 cases in our cohort had a BMI ≥28 kg/m^2^, limiting the assessment of the impact of severe obesity, which has been previously linked to increased CD hospitalization risk through exacerbated intestinal barrier damage (19). Crucially, the observed “protective” effect of BMI in this study applies only to underweight or normal-weight CD patients and should not be generalized to obese populations. Simultaneously, the observed “protective” association of BMI should be interpreted cautiously within the dynamic context of weight change in IBD. Evidence indicates that weight gain in IBD patients, particularly exceeding 6%, is a risk factor for the progression of hepatic steatosis (20). This suggests that while a higher BMI within a certain range may indicate better nutritional reserves to counter inflammatory catabolism, unregulated weight gain, especially during disease remission, may introduce new metabolic co-morbidities such as MASLD, potentially complicating the long-term disease course. The use of BMI instead of waist circumference to define the obesity component in our study warrants further discussion. Waist circumference is widely recognized as the gold standard for assessing visceral obesity and central fat distribution, which has a more direct relationship with insulin resistance and meta-inflammation than BMI (21). This distinction may be particularly relevant in CD patients. CD often leads to muscle wasting and weight loss, potentially resulting in a “normal-weight obesity” phenotype, where patients have a normal BMI but a disproportionately high level of visceral fat. In such cases, reliance on BMI may fail to identify these individuals at risk of metabolically adverse outcomes driven by visceral adiposity. Recent studies emphasize that waist circumference holds the highest predictive value for identifying concomitant MASLD in IBD patients (21). However, the systematic availability and completeness of waist circumference data are common challenges in large retrospective cohorts. The Chinese MetS diagnostic criteria adopted in our study permit the use of BMI, ensuring feasibility and standardization, but we acknowledge that this approach may have attenuated our ability to detect associations specifically linked to central obesity. Nonetheless, the significant associations we observed between BMI and nutritional status or disease activity underscore the critical importance of overall energy reserves in CD. Future prospective studies incorporating precise measures of body composition, such as waist circumference and bioelectrical impedance analysis, are warranted to dissect the specific roles of visceral versus subcutaneous adipose tissue in modulating CD outcomes. A Mendelian randomization analysis identified a causal association between hypertension and an increased risk of inflammatory bowel disease (IBD) (OR = 2.32, *p* = 0.028) (11), but we found no significant association between hypertension and poor CD outcomes. This discrepancy may stem from differences in the study populations: the former focused on the risk of IBD incidence, whereas our study focused on the outcomes in already diagnosed CD patients, suggesting that the role of hypertension may differ across disease stages.

One of the most significant findings of this study reveals a distinct “dose–response” effect of metabolic syndrome elements—both disease severity and the risk of adverse clinical outcomes in CD exhibited a linear increase with a higher number of metabolic abnormalities. The underlying mechanisms are likely multifactorial, centering on the synergistic amplification of “meta-inflammation.” Firstly, aggravated systemic low-grade inflammation serves as a central link between MetS and CD. Visceral adiposity, hyperglycemia, and dyslipidemia contribute to persistent immune activation through distinct pathways, including reduced adiponectin secretion and increased pro-inflammatory cytokine production from adipose tissue, hyperglycemia-promoted oxidative stress and the generation of advanced glycation end products, and stimulation by free fatty acids and oxidized low-density lipoprotein (6, 11, 22). When these pathways converge in an individual, they likely act synergistically, fostering a more intense and recalcitrant systemic inflammatory state that lowers the threshold for intestinal inflammation and exacerbates tissue damage. Secondly, cumulative impairment of the intestinal barrier is a critical factor. Individual components of MetS have been shown to damage epithelial tight junctions, alter mucus composition, and disrupt gut microbiota homeostasis (23). The coexistence of multiple metabolic defects may deliver “multiple hits” to the intestinal barrier, leading to more severe increases in intestinal permeability, enhanced translocation of microbial antigens, and persistent stimulation of the mucosal immune system, ultimately resulting in refractory disease activity. Therefore, clinical assessment should extend beyond the binary diagnosis of MetS; counting the number of abnormal metabolic elements is essential, and even the presence of 1–2 components should be considered a risk signal. It is worth noting that recent research has begun to explore the use of metabolic scores and lipid ratios to quantify the metabolic burden in IBD patients and predict the risk of complications such as MASLD. For instance, metrics like the metabolic score for insulin resistance, lipid accumulation product, and the triglyceride-to-high-density lipoprotein cholesterol ratio have been demonstrated to effectively predict the onset of MASLD in IBD patients (21). This provides external validation for our “cumulative number” effect and suggests potential clinical applications. These scores essentially integrate multiple metabolic abnormalities into a single composite measure, which aligns well with our finding that a higher number of MetS components is positively associated with an increased risk of adverse CD outcomes. In the long-term management of CD, regular screening of these metabolic indicators and calculation of relevant risk scores may facilitate the early identification of patients with a high metabolic burden, who are consequently at risk for more severe CD progression and hepato-intestinal co-morbidities.

The potential influence of medications for metabolic disorders requires careful consideration. Although our core metabolic and disease activity indicators were collected prior to the initiation of CD-specific treatment, thereby reducing direct confounding by these therapies, we cannot entirely exclude the influence of medications that patients might have been taking for pre-existing conditions such as hypertension, diabetes, or dyslipidemia (e.g., statins, antihypertensive agents, hypoglycemic drugs). For instance, statins are suggested to possess anti-inflammatory and immunomodulatory properties beyond their lipid-lowering effects (24), and certain antihypertensive drugs may also modulate intestinal inflammatory pathways (25). The use of such medications could alter baseline metabolic parameters and the systemic inflammatory state, potentially confounding the true association between MetS components and CD outcomes. Future well-designed prospective studies that systematically collect and analyze medication history are needed to further clarify the causal relationships.

A major strength of this study is its systematic evaluation of the associations between all MetS elements and CD, coupled with a stratified analysis based on the number of coexisting MetS elements, thereby providing a comprehensive assessment of the cumulative impact of metabolic abnormalities. Limitations include inherent biases of the retrospective design; the lack of reliable medication history limited our ability to control for the effects of these drugs on metabolic measures and disease progression; the use of BMI instead of waist circumference may underestimate the impact of central obesity and lack of longitudinal data to establish causal sequence.

## Conclusion

The presence and cumulative number of MetS elements are independently associated with more severe disease activity and worse clinical outcomes in CD, demonstrating a clear additive effect. Low HDL-C is the most prominent risk factor. Screening and management of metabolic abnormalities, particularly dyslipidemia, may improve long-term prognosis in CD. These findings warrant validation in prospective studies.

## Data Availability

The original contributions presented in the study are included in the article/Supplementary material, further inquiries can be directed to the corresponding author.
