# Supplementary material for: Individual components and cumulative burden of metabolic syndrome are associated with higher disease activity and adverse outcomes in Crohn’s disease
Source: Front Med (Lausanne). 2025 Dec 16;12:1721566. doi: 10.3389/fmed.2025.1721566 (PMC12748238; doi:10.3389/fmed.2025.1721566)
Supplement: Supplementary file 1 [file Table_1.docx]

**Supplementary table 1** Relationship between metabolic syndrome elements and laboratory indicators, clinical scores, and hospitalization characteristics in Crohn's disease, median [IQR].

| Outcomes | Low HDL-C | Non-  Low HDL-C | *P-*value | HTG | Non-HTG | *P-*value | Hyperglycemia | Non-  Hyperglycemia | *P-*value | Hypertension | Non-  Hypertension | *P-*value |
| --- | --- | --- | --- | --- | --- | --- | --- | --- | --- | --- | --- | --- |
| ESR, mm/h | 27 (15, 44) | 17 (8, 27) | ＜0.001^a^ | 25 (12, 44) | 23 (12, 38) | 0.43 | 7 (13, 53） | 23（12, 37） | 0.167 | 23（12, 43） | 23（12, 37） | 0.383 |
| Serum albumin, g/L | 35.10  (30.1, 38.6) | 38.80  (35.2, 42.3) | ＜0.001^a^ | 36.40  (32.18,40.08) | 36.60  (31.40,39.9) | 0.64 | 34.95  (29.10,37.55） | 36.70（31.85,40.00） | 0.095 | 36.60（32.90,40.50） | 36.50（31.10,39.70） | 0.224 |
| Serum prealbumin, mg/L | 148.20  (107.60,209.20) | 195.10  (154.60,254.20) | ＜0.001^a^ | 191.70  (132.10,265.50) | 159.30（119.20,214.10） | 0.003^a^ | 123.50  (94.40,185.40) | 168.80  (122.10,229.30) | 0.031^a^ | 184.00  (129.80,257.70) | 162.60（117.10,220.30） | 0.025^a^ |
| Serum ferritin, ug/L | 232.50  (89.83,358.90) | 164.80  (71.00,250.70) | 0.007^a^ | 241.30  (85.57,456.10) | 188.80  (78.27,305.10) | 0.047^a^ | 268.00  (108.20,759.60) | 190.40  (78.37,321.40) | 0.067 | 253.20  (123.50,440.10) | 185.60  (73.24,305.10) | 0.008^a^ |
| Fecal calprotectin,  μg/g | 631.40  (449.80,843.70) | 398.50 (187.80,686.70) | ＜0.001^a^ | 660.40  (458.80,1022.00) | 557.80  (338.00,745.00) | 0.012^a^ | 733.00  (548.30,1116.00) | 582.90  (341.30,798.40） | 0.057 | 612.30  (420.10,1075.00) | 582.90  (338.20,790.90) | 0.278 |
| SES-CD | 8（6,10） | 6（4,9） | ＜0.001^a^ | 8(6, 10) | 6(4, 7) | ＜0.001^a^ | 10 (7, 13) | 7（5, 10） | 0.003^a^ | 8（6, 12） | 7 (5, 10) | 0.011^a^ |
| CDAI | 274.20  (239.40,310.00) | 220.00  (188.80,253.20) | ＜0.001^a^ | 282.00  (240.10,320.00) | 256.00  (218.60,290.00) | ＜0.001^a^ | 290.90  (245.90,342.60) | 256.00  (220.00,296.90) | 0.037^a^ | 258.70  (220.40,317.60) | 256.20  (220.00,294.20） | 0.278 |
| Age, years | 31（22, 44） | 33（24, 50） | 0.11 | 40（27, 54） | 30（22, 41） | ＜0.001^a^ | 46（28, 51） | 31（23, 44） | 0.022^a^ | 41（29, 58） | 30（22, 41） | ＜0.001^a^ |
| Length of hospital stay, days | 9（8, 13） | 8（7, 12） | 0.002^a^ | 11（8, 14） | 9（7, 12） | 0.002^a^ | 9（7.25, 12.75） | 9（7, 12） | 0.005^a^ | 10（7, 13） | 9（7, 12） | 0.191 |
| Total costs, thousand CNY | 14.30  （11.54, 20.01） | 11.45  （9.08, 14.45） | ＜0.001^a^ | 17.35  (13.52,24.80) | 13.06  (10.20,16.89) | ＜0.001^a^ | 17.92（12.51,40.70） | 13.58（10.48,18.01） | 0.644 | 15.51  （11.80, 19.40） | 13.26（10.36,17.89） | 0.027^a^ |

^a^P < 0.05;

IQR, interquartile range; Low HDL-C, low high-density lipoprotein cholesterol; HTG, hypertriglyceridemia; ESR, erythrocyte sedimentation rate; SES-CD, simplified endoscopic score for Crohn's disease; CDAI, Crohn's Disease Activity Index; CNY, Chinese Yuan.

The Mann–Whitney U test was used for continuous variables.
